# H-FABP as a diagnostic marker for early detection of young myocardial infarction among Indians

**DOI:** 10.6026/97320630018506

**Published:** 2022-06-30

**Authors:** Anoop Jaiswal, Amrita Vamne, Manish Kumar Verma, Beenu Doctor

**Affiliations:** 1 Department of Biochemistry Index Medical College, Malwanchal University Indore, India; 2 Department of Biochemistry, GSVM Medical College, Kanpur, India; 3 Department of Biochemistry, Moti Lal Nehru Medical College, Prayagraj, India

**Keywords:** Human heart-type fatty-acid-binding protein, CK-MB, Troponin T, high-sensitivity C-reactive protein, Young myocardial infarction

## Abstract

It is of interest to document the point-of-care test using heart-type fatty-acid binding protein (H-FABP) in comparison with CK-MB, Troponin T and hsCRP. This is a more sensitive and specific cardiac biomarker than cTnT and CK-MB, and it has a higher diagnostic effectiveness for detecting early acute myocardial infarction (AMI). The case-control study enrolled 220 participants (110 myocardial infarction patients as cases and 110 healthy subjects as control) > 18 years of either sex after ethical clearance and informed consent form. The study conducted was conducted in the OPD and IPD of Medicine and Biochemistry Department at Moti Lal Nehru Medical College and Swaroop Rani Nehru Hospital Prayagraj, Uttar Pradesh; Index Medical College & Hospital, Malwanchal University, India. The amount of H-FABP, CKMB and cTnT was measured using the Sandwich ELISA method and hs-CRP was evaluated using the immune-turbidimetry method. H-FABP correlation with selected markers (CK-MB, hs CRP and TnT) and CK-MB was significant. A positive correlation (r=0.2 to 0.29) was found when H-FABP was compared with CK-MB (p<0.05). Similar positive correlation was found in CK-MB with cTnT. H-FABP is a useful cardiac marker for the early diagnosis of young AMI and thus prediction of myocardial injury is possible. H-FABP compared with CK-MB showed positive correlation. CK-MB with cTnT also showed statistically significant relation. Thus, H-FABP and CK-MB, as well as the correlation between CK-MB and TnT, reflects utility in early-stage diagnosis of myocardial injury.

## Background:

Acute myocardial infarction (AMI) is a main life-threatening cardiac condition for which early and accurate diagnosis is critical in order to allow for urgent and intensive treatment, which minimises mortality [1]. Chest discomfort is one of the most prevalent and serious symptoms among patients who visit the Emergency Department. Other common causes of chest pain (CP) include gastro-oesophageal reflux disease, musculoskeletal ailments, and pulmonary diseases [2]. Acute myocardial infarction (AMI) accounts for 5-15 percent of all causes of CP. In India, a significantly higher number of patients report to the emergency department with CP as their primary complaint [3]. 40 percent of patients presenting with acute CP could be spared the dangers and costs of unwarranted hospital admission and more invasive cardiac testing if cardiac pain could be reliably ruled out [4]. AMI mortality and long-term morbidity can be reduced if diagnosed and treated correctly early. The majority of AMI-related deaths occur within the first hour of start of symptoms. If AMI cases are detected and treated well during the first hour (the "Golden Hour") after start of symptoms, death can be decreased from 9% to 3%, but if diagnosed and treated after 3-4 hours, mortality can be five times greater [5]. AMI is commonly diagnosed using the creatine kinase–MB isoenzyme (CK-MB), myoglobin, and cardiac troponins (cTnI & cTnT). These cardiac marker proteins, on the other hand, are insufficient for diagnosing AMI during the first 6 hours of the beginning of chest pain. Myoglobin, which occurs in the blood within two hours of a myocardial infarction, lacks specificity because myoglobin released from skeletal muscles and that released from the heart cannot be differentiated. Although cardiac troponins and CK-MB are more specific for heart damage, their blood concentrations do not rise until 6-8 hours after the onset of symptoms, limiting their early sensitivity [6]. H-FABP (heart-type fatty acid binding protein) is a new marker that has the potential to detect AMI within 6 hours after beginning of symptoms. Compared to establish cardiac biomarkers, it has a number of theoretical advantages. H-FABP is a 132-amino-acid soluble protein with a molecular weight of 15 kDa. In cardiomyocytes, it is one of the most abundant proteins, accounting for 5-15 percent of the total cytosolic protein pool. It aids in the transport of fatty acyl coenzyme A to the mitochondria for oxidation. H-FABP is not found in plasma under normal circumstances. H-FABP leaks out of cardiac tissue during ischemia, and its concentration rises in the blood within 2 hours, peaking at around 4-6 hours and returning to normal baseline value in 20 hours [7]. H-FABP is a more sensitive and specific cardiac biomarker than myoglobin and CK-MB, and it has a higher diagnostic effectiveness for detecting myocardial infarction within 6 hours after experiencing chest pain. H-FABP can be utilized as a supplement to other tests for the early diagnosis of myocardial infarction 8]. Therefore, it is of interest to study the correlation of H-FABP with CKMB, hs-CRP and cTnT in young myocardial infarction patients.

## Materials and methods:

In this case-control study 220 (110 case and 110 control) participant of Acute Coronary Syndrome patients (either sex) taken and the patients would be identified according to the standardized methodology and a pre-designed proforma and study conducted in the OPD and IPD of Cardiology or Medicine department at Index Medical College & Hospital, Malwanchal University and Moti Lal Nehru Medical College and Swaroop Rani Nehru Hospital Prayagraj in India. The entire participants were enrolled after the informed written consent form.

### Inclusion criteria:

1) Patients more than 18 years of age, ECG findings and biochemical markers

2) Suggestive of acute myocardial infarction.

3) Elevated value of CK-MB and TnT

4) Chest pain lasting 24 hours, suggestive of myocardial ischemia of accelerated pattern, or a prolonged one (> 20 minutes), or with recurrent episodes at rest, or at minimal exertion.

### Exclusion criteria:

1) Known causes of elevated uric acid level (chronic kidney disease, gout, hematological malignancy, and hypothyroidism).

2) Patients on drugs which increase serum uric acid e.g., salicylates (2gm/dl, hydrochlorothiazide, pyrazinamide).

3) Chronic alcoholics.

4) Acute phase of impaired subject of obesity (body mass index > 30) will be excluded. In addition, patients receiving medications affecting lipid metabolism, such as lipid lowering drugs, beta-blockers, oral contraceptives, estrogen, progestin, thyroxin and vitamin E will be also excluded.

5) Present or past aspirin, statins or hormone replacement therapy, autoimmune diseases and malignancies smokers, Subjects with any chronic diseases or acute infections, antioxidant vitamin supplements, hepatic disease etc.

### Sample collection: 

The fasting blood samples were collected venous blood samples were centrifuged on 4000 RPM for 5 minute and stored at -80°C in a deep freezer until being analyzed.

### Evaluation of Biomarkers:

H-FABP, CKMB and cTnT by Sandwich ELISA Method and hs-CRP was evaluated by immunoturbidimetry method.

### Statistical Analysis:

For continuous data, normality was tested using Kolmogorov Smirov test. For non-normal continuous data, χ^2^ test, Mann Whitney U test were used to compare the groups as appropriate. Categorical data were presented in frequency and percentage. For age, Uni variate binary logistic regression analysis was used to calculate unadjusted odds ratio (OR) and 95% confidence interval (CI) in different variables between case control. Baseline and principal data were compared between case and controls using 2 x 2 contingency table calculator available online at (http://faculty.vassar. edu/lowry/VassarStats.html. Statistical analysis was carried out using the statistical package for the social science, version 22 (SPSS-22, IBM, Chicago, USA). Two tailed p value < 0.05 has been considered as significant.

## Results:

### Distribution of gender characteristics on the basis of principal characteristics of cases and controls: 

In this distribution 110 cases and 110 controls are subcategorized into male and female which were shown in Table 1(see PDF). Results revealed that in subgroup analysis of case having male verses female was not found to be null in all selected parameters (all p>0.05). This result was similar in control group also (all p>0.05). In other words, subjects of two groups are same when compared between male and female in specific group. But when significance was calculated between case and control through unpaired T test then different scenario observed. All the selected biomarkers were found significantly high in case than its control group (all p <0.05).

### Correlation between selected biochemical markers:

The biochemical marker levels of the two groups are summarized in Table 2(see PDF) and also shown in Figure 1 to 2(see PDF). Comparing the mean biochemical parameter levels of the two groups, Student’s t test showed significant difference (p<0.01). The mean hsCRP (mg/L) and FABP (ng/ml) were found significantly different and almost 8 and 5-fold higher in cases as compared to controls (4.62 ± 1.49 vs. 0.54 ± 0.6, F=107.5; t=28.6 and 13.25 ± 4.04 vs 2.29 ± 0.44; F=161.3.5; t=28.1; p<0.01). Further, the CK-MB (IU/L) and cTnT (pg/ml) (mean ± SD) were also high in case group than control (166.9 ± 26.7 vs. 98.6 ± 3.1, F=131.4; t=26.8; p<0.01 and 134.7 ± 8.57 vs 96.6 ± 2.82; F=145.9; t=44.3; p<0.01). The comparisons concluded that selected all markers may have an influence on cases.

### Correlation between selected biochemical markers: 

Correlation coefficient (r) is defined as a statistical measurement of the interdependence of two or more random variables. Correlation analysis measures the closeness and degree of linear association between independent and dependent variables. Correlation was found to be null between all selectable markers except FABP with CK-MB. A positive correlation (r=0.2 to 0.29) was found when compare FABP & CK-MB (p<0.05) similarly positive correlation was found in CK-MB with cTnT. The relation of these selected markers was shown in Table 3(see PDF) is visualized in Figure 1 and 2(see PDF).

## Discussion:

In this case control study aimed to investigate the correlation of H-FABP with CKMB, hs-CRP and cTnT in young Myocardial Infarction Patients. In recent study acute myocardial infarction is the leading cause of death and long-term morbidity. Early and accurate diagnosis is critical because it allows for quick and intensive therapy, which lowers mortality [9]. Based on the varied circulatory release timings of cardiac markers following myocardial injury, some studies postulated that H-FABP would be useful in the early detection of AMI [10-14]. Although there is no consensus on the diagnostic value of H-FABP, some studies have suggested that it may be useful in the early detection of AMI and the prediction of MI [15,16]. The gold standard for the biochemical diagnosis of acute MI is cardiac troponins I and T, which are used in conjunction with clinical symptoms and ECG abnormalities [17, 18]. Troponins are big proteins found mostly inside the cardiac fibre structure.

Because of troponin's enormous size and location, detecting serum levels of troponin in patients with an acute MI can take several hours [19]. The most often utilized cardiac markers for the identification of AMI are cardiac troponins T, CK-MB, and hsCRP tests [20]. Collisson et al. demonstrated that AMI diagnosis cannot be achieved only on the basis of individual CK-MB or troponin I test results [21]. In our study was CK-MB and cTnT (mean±SD) were also high in case group than control (p<0.01). The comparisons concluded that selected all markers may have an influence on cases. Further the mean hsCRP and FABP were found significantly different and almost 8 and 5-fold higher in cases as compared to controls (p<0.01). In a previous study by J Rosman et al 2009. Peak troponin levels linked with qualitative and quantitative H-FABP in 40 patients who had a MI. The percentage of positive, qualitative H-FABP assay results (Cochran-Armitage test for trend, p 0.05) and quantitative H-FABP values (Cochran-Armitage test for trend, p 0.001) increased as the peak level of troponin increased. The accuracy of first troponin and H-FABP in the early identification of acute MI was compared in patients who reported within 1 hours of the beginning of chest pain. H-FABP, both qualitative and quantitative, was less sensitive than initial troponin I & T [22]. Present study correlation of H-FABP with selected markers (CK-MB, hs CRP,and TnT) only with CK-MB significant association found. A positive correlation (r=0.2 to 0.29) was found when H-FABP compare with CK-MB (p<0.05) similarly positive correlation was found in CK-MB with cTnT. Previous study was O'Donoghue M et al 2006, also found statistically significant correlations (r=0.29, p<0.001) in Table 3(see PDF) [12]. Thus, the correlation between H-FABP and CK-MB may reflect significant differences in release thresholds and circulating level kinetics. The sensitivity of troponin and CK-MB in detecting early reinfarction or low-grade ischemia may be limited by slower plasma clearance and troponin and CK-MB rise times. H-FABP, on the other hand, is a smaller molecule that is heavily concentrated in the cytoplasm of cardiomyocytes and is swiftly removed, making it a better candidate for detecting minor recurring cardiac damage [12]. Pyati et al. 2015 found that mean blood concentrations of H-FABP, hsCRP, and CK-MB activity were substantially greater (p 0.01) in AMI cases compared to controls in a prior study [8]. In addition, when compared to the 0–3-hour group of AMI cases, the mean values of cardiac markers were substantially greater in the 3-6-hour group. This result is consistent with the findings of Glatz JFC et al., Elmadbouh I et al., Pasaoglu H et al., and Orak M et al. [23-26]., Anand K Pyati et al. 2015, shown that earlier rise in serum concentration of H-FABP compared to myoglobin and CK-MB in AMI is likely due to its smaller molecular size (15kDa and 17kDa for H-FABP and myoglobin, respectively), higher concentration in myocardial tissue (concentrations of H-FABP concentrations are 2-10 fold higher in heart than skeletal muscle) a Despite the fact that H-FABP is not entirely cardiac specific, its tissue distribution outside of the heart is equivalent to that of CK-MB [8]., Finally, Rosman et al. 2009 demonstrated that elevated serum H-FABP plays a critical role in the early detection of acute coronary syndromes. H-FABP was found to be highly sensitive and specific in a single measurement, which could lead to speedier triaging of emergency patients and earlier discharge of individuals with non-cardiac chest pain. They also demonstrated that H-FABP not to be a sensitive diagnostic for the early diagnosis of acute MI. Troponin exhibited a higher diagnostic yield than H-FABP, especially in the early hours after symptom onset. As a result, its utility as an acute coronary syndrome biomarker is restricted [22]. J cornor et al. 2008, shown that H-FABP, in addition to cTnT, should be measured at the time of admission in patients with acute ischemic chest pain to aid in the early identification of acute MI. H-FABP has a much higher sensitivity than cTnT for individuals who present within 4 hours of illness onset. A combination strategy boosts sensitivity even further for some patients. When H-FABP is measured with cTnT at the time of admission, there is a positive rise in negative predictive value, implying that H-FABP can help rule out acute MI 27]. H-FABP is a very sensitive biomarker for individuals with early chest discomfort for the early identification of AMI [28]. Thus, H-FABP is a promising cardiac biomarker to be utilized in conjunction with troponins and CK-MB [29, 30].

## Conclusion:

Point-of-care test of H-FABP was a more sensitive and a prognostic test for young AMI diagnosis compared to CK-MB, hs CRP and TnT. A positive correlation (r=0.2 to 0.29) was found for H-FABP with CK-MB (p<0.05). Moreover, a positive correlation was found in CK-MB with cTnT. Thus, the correlation between H-FABP and CK-MB, as well as the correlation between CK-MB and TnT, reflects differences in release thresholds and circulating level. The sensitivity of troponin T and CK-MB in detecting early reinfarction or low-grade ischemia may be limited by slower plasma clearance with troponin T and CK-MB raise times. H-FABP, on the other hand, is a smaller molecule that is heavily concentrated in the cytoplasm of cardiomyocytes and is swiftly removed, making it a better candidate for detecting minor recurring cardiac damage.
